# A Cluster of Meningococcal Serogroup W135 Infections: A Case Series

**DOI:** 10.7759/cureus.80747

**Published:** 2025-03-17

**Authors:** Usamah Al-Anbagi, Muna Al Maslamani, Abdullatif Al-khal, Mohamed Mohamedali, Muayad K Ahmad, Sulieman Abu Jarir, Manal Hamed, Elsa Suby, Moustafa S Elshafei, Dore Chikkahanasoge Ananthegowda, Mas Chaponda, Hamad E Al-Romaihi, Abdulqadir J Nashwan, Mohamed Aboukamar

**Affiliations:** 1 Internal Medicine, Hamad Medical Corporation, Doha, QAT; 2 Infectious Diseases, Hamad Medical Corporation, Doha, QAT; 3 Medicine, Hamad Medical Corporation, Doha, QAT; 4 Laboratory Medicine and Pathology, Hamad Medical Corporation, Doha, QAT; 5 Microbiology, Hamad Medical Corporation, Doha, QAT; 6 Critical Care, Hamad Medical Corporation, Doha, QAT; 7 Epidemiology and Public Health, Ministry of Public Health, Doha, QAT; 8 Nursing & Midwifery Research, Hamad Medical Corporation, Doha, QAT; 9 Infectious Disease, Hamad Medical Corporation, Doha, QAT

**Keywords:** acute respiratory distress syndrome (ards), fever, meningococcal meningitis, meningococcemia, myocarditis, neisseria meningitidis (n. meningitidis), purpura fulminans, rhabdomyolysis, septic shock, serogroup w135

## Abstract

*Neisseria meningitidis* serogroup W135 is associated with a broad spectrum of clinical manifestations, ranging from mild localized infections to life-threatening systemic diseases. This report presents four cases of meningococcal infection in Qatar with varying presentations and complications. All patients were previously healthy, with no comorbidities, and had not received the meningococcal vaccine. All cases demonstrated elevated inflammatory markers, including C-reactive protein (CRP) and procalcitonin, and evidence of organ dysfunction, such as acute kidney injury, rhabdomyolysis, and myocarditis. Treatment with broad-spectrum antibiotics (ceftriaxone and meropenem) and aggressive supportive care, including vasopressors, mechanical ventilation, and continuous renal replacement therapy, led to gradual improvement in all patients. These cases underscore the importance of early recognition, prompt antibiotic therapy, and close monitoring of prognostic indicators, such as thrombocytopenia, elevated D-dimer, and lactate levels, in managing meningococcal disease. The variability in clinical presentations highlights the need for heightened clinical suspicion and tailored management strategies to improve outcomes in this potentially fatal condition.

## Introduction

*Neisseria meningitidis*, a gram-negative diplococcus, is a leading cause of bacterial meningitis and septicemia worldwide [1]. Despite advances in vaccination and antibiotic therapy, meningococcal disease remains a significant public health concern due to its rapid progression, high morbidity, and mortality rates [2]. The clinical spectrum of *N. meningitidis* infection is broad, ranging from asymptomatic nasopharyngeal colonization to severe invasive disease, including meningitis, meningococcemia, and septic shock [2]. Serogroup W135, particularly the hypervirulent ST-11 clonal complex, has been associated with outbreaks and severe disease among travelers and pilgrims [3].

The pathogenesis of meningococcal disease involves nasopharyngeal colonization, invasion of the bloodstream, and systemic dissemination, leading to endotoxin-mediated inflammation, coagulopathy, and multiorgan failure [3]. Early recognition and prompt treatment are critical, as delays in antibiotic therapy and supportive care can result in poor outcomes, including death or long-term sequelae such as neurological deficits, limb loss, and hearing impairment [3].

This case report highlights four patients with *N. meningitidis* serogroup W135 infection reported in Qatar, each presenting with distinct clinical manifestations and complications. These cases illustrate the diverse spectrum of meningococcal disease, from localized infections to life-threatening systemic illness, and underscore the importance of early diagnosis, aggressive management, and close monitoring of prognostic indicators. Enhancing awareness of the varied presentations of meningococcal disease and emphasizing the need for timely intervention to improve patient outcomes is crucial.

## Case presentation

Case 1

A previously healthy 30-year-old male, who had not received the meningococcal vaccine, returned to Saudi Arabia one day before symptom onset after performing Umrah. His illness began with fever, diffuse body aches, sore throat, abdominal pain, nausea, vomiting (five episodes), multiple episodes of diarrhea, mild shortness of breath, and significant fatigue. He denied having any cough or chest pain.

On admission, he was found to be febrile (temperature >39°C), hypotensive 74/47 mmHg, and tachypneic 21 breaths/min. Despite receiving over 3 liters of fluid resuscitation, his blood pressure remained low, necessitating the initiation of vasopressors (dopamine and noradrenaline). Initial physical assessment in the emergency department (ED) indicated severe dehydration without signs of meningitis. He was started on piperacillin/tazobactam 4.5 g, and the medical ICU team was consulted due to worsening hypotension and rising lactate levels.

**Figure 1:**
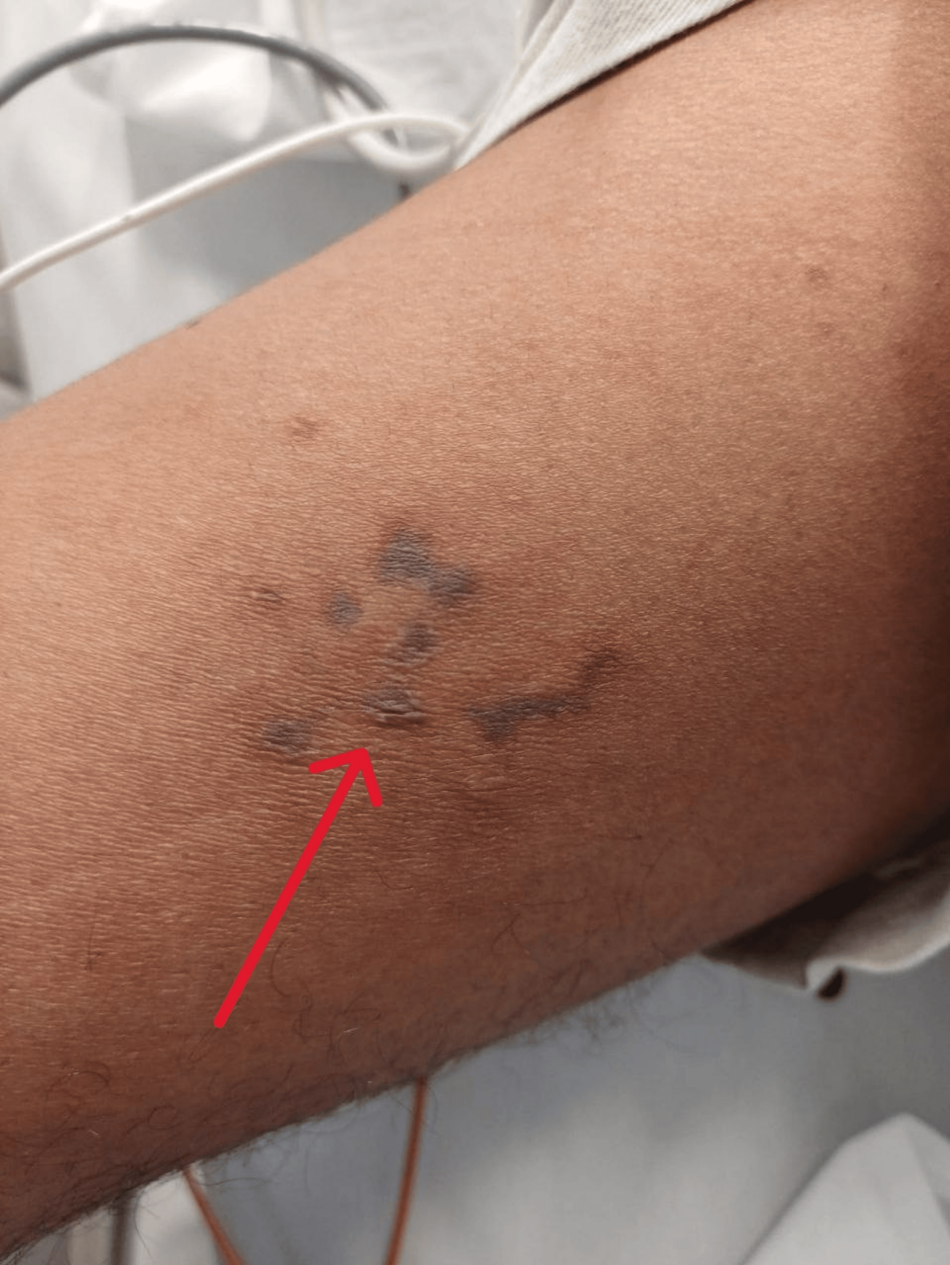
Meningococcal purpura fulminans rash

Upon ICU admission, he remained febrile (39.3°C) with tachycardia (heart rate 122 bpm), tachypnea (respiratory rate 20 breaths/min), and persistent hypotension (blood pressure 75/48 mmHg), requiring continued vasopressor support. A focused physical examination revealed marked dehydration, purpuric rash on the shins and forearms, palatal petechiae, poor inspiratory effort, and cold extremities with palpable pulses (Figure 1). Laboratory investigations showed an increased white blood cell count, elevated procalcitonin, high D-dimer, significantly elevated troponin T, and high lactate levels (Table 1).

**Figure 2:**
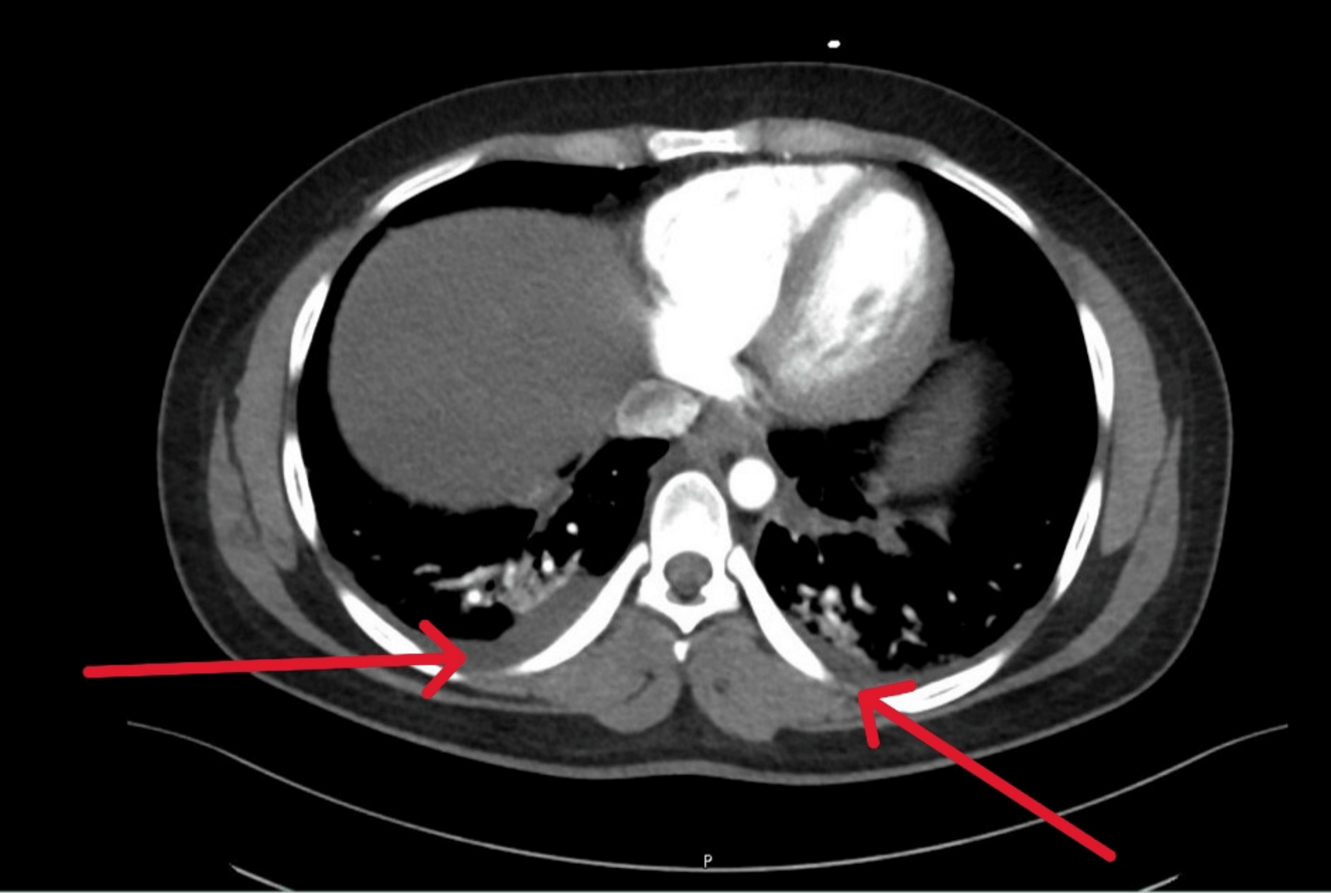
Minimal bilateral pleural effusions with bibasilar dependent atelectasis/consolidation

A chest X-ray showed no overt pulmonary findings. A CT angiogram of the abdomen performed to rule out mesenteric ischemia showed minimal bilateral pleural effusions with bibasilar atelectasis/consolidation and subtle ground-glass opacities (Figure 2). An echocardiogram demonstrated an ejection fraction (EF) of 41%, mild global left ventricular hypokinesis, mild mitral regurgitation, and mildly elevated pulmonary artery pressure. The patient also developed atrial fibrillation, and a cardiology consultation confirmed acute myocarditis secondary to meningococcal infection. He was started on aspirin, oral anticoagulation with rivaroxaban 20 mg daily, and bisoprolol 2.5 mg.

**Figure 3:**
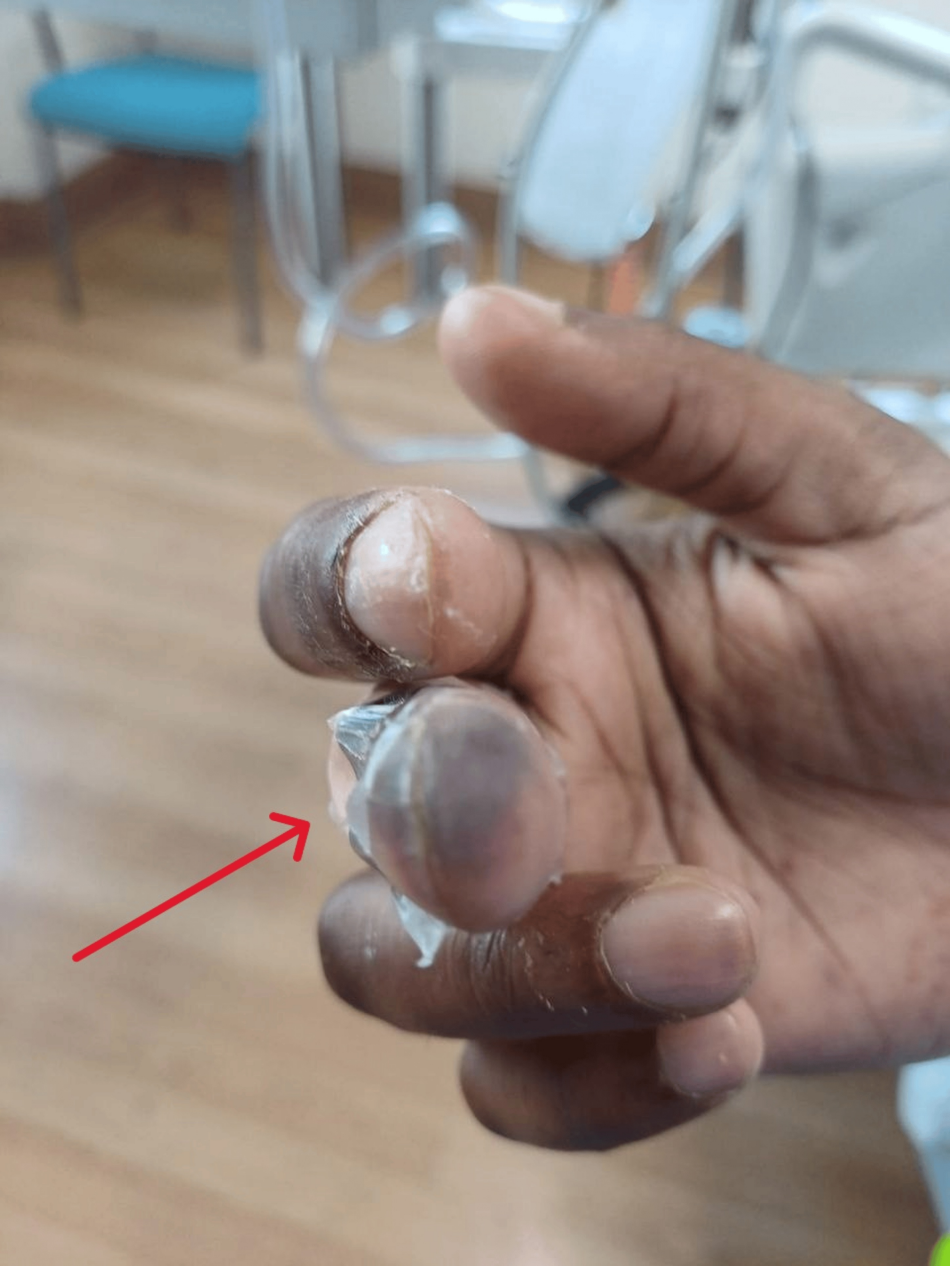
Violaceous discoloration of the distal phalanx of the right middle finger with epidermal separation, suggestive of necrosis, consistent with purpura fulminans

The patient was diagnosed with septic shock and possible acute myocarditis. Noradrenaline was continued, and antibiotic therapy was escalated to meropenem 2 g every eight hours. Dexamethasone 1 mg intravenous every six hours was initiated. On the second day, he developed blackish discoloration of the right middle finger (Figure 3) and second toe, raising concern for peripheral ischemia due to septic shock.

**Figure 4:**
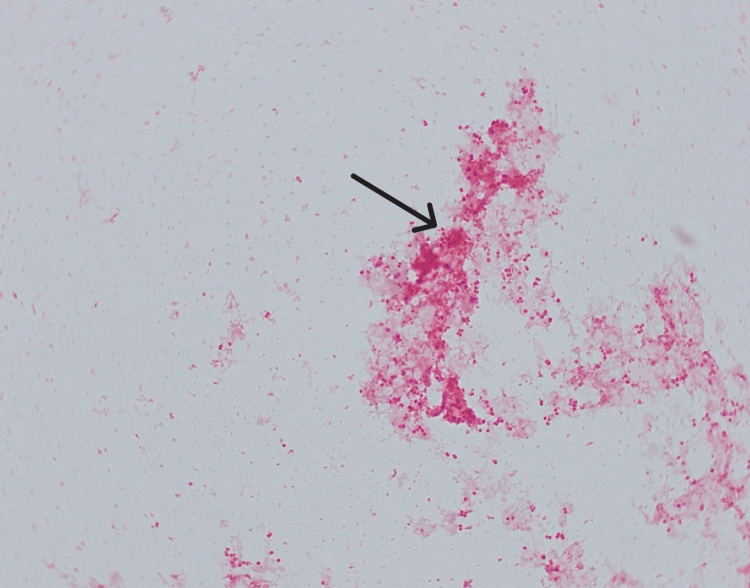
Gram negative diplococci from direct gram stain of the blood culture positive

Inflammatory markers continued to rise, with the WBC count and C-Reactive Protein (CRP) levels increasing. On the third day, blood cultures confirmed *Neisseria meningitidis* serogroup W135 (Figure 4). Meningitis was suspected, but the patient refused a lumbar puncture. Infectious disease specialists recommended continuing meropenem.

By the fourth day, vasopressors were successfully discontinued, and the patient showed clinical improvement. He was stepped down to the medical floor, where, based on sensitivity results, antibiotics were de-escalated to ceftriaxone 2 g twice daily. The atrial fibrillation was resolved, and he returned to sinus rhythm. He continued to improve clinically and was discharged after completing two weeks of antibiotics. He was discharged on rivaroxaban 20 mg and bisoprolol 2.5 mg, with an appointment given for follow-up.

Case 2

A previously healthy 27-year-old male, who had not received the meningococcal vaccine, was brought in by ambulance after experiencing multiple generalized tonic-clonic seizures without regaining consciousness between episodes. According to his roommate, the patient had been experiencing fever, generalized weakness, and fatigue for the past two days. Upon arrival at the ED, the patient was responsive to voice but had defecated in bed and was in a postictal state. During this time, one seizure episode was observed. According to bystanders, the patient had no prior history of epilepsy and was not on any regular medications. Another seizure episode was then witnessed, prompting intubation at the scene. He was administered 4.5 grams of levetiracetam and started on a midazolam infusion for seizure control.

Physical examination revealed a Glasgow Coma Scale (GCS) of 6T (E1, V1, M6), with reactive but sluggish pupils (4 mm). The chest was clear on auscultation, and the patient was on mechanical ventilation with a PaO₂/FiO₂ ratio of 25%. Cardiovascular examination revealed normal heart sounds (S1/S2), and the abdomen was soft and non-tender. No rash or petechiae were noted on the skin.

Laboratory investigations revealed an increased WBC count, decreased platelets, decreased hemoglobin, and a markedly increased D-dimer, consistent with disseminated intravascular coagulation. Chemistry results showed an increased creatine kinase, consistent with rhabdomyolysis, an increased CRP, and an increased procalcitonin level (Table 1).

**Table 1:** Laboratory results MCV: Mean Corpuscular Volume, MCH: Mean Corpuscular Hemoglobin, AST: Aspartate Aminotransferase, ALT: Alanine Aminotransferase, CK: Creatine Kinase, CRP: C-Reactive Protein, PT: Prothrombin Time, INR: International Normalized Ratio, APTT: Activated Partial Thromboplastin Time

Cases	Case 1			Case 2			Case 3			Case 4			Reference values
Days	Day 1	Day 3	Day 7	Day 1	Day 3	Day 7	Day 1	Day 3	Day 7	Day 1	Day 3	Day 7	
Total leukocytes	25	43	33	12.5	12	9	17.6	22.8	17.8	19	56	39	(6.2 x10^3^/uL)
Hematocrit	37.2	40	13	36.9	35	33	39.6	31	27.9	40	26.8	19.7	(40-50%)
Hemoglobin (gm/dL)	12.9	13.5	40	12.6	12.2	11.4	13.8	10.3	9.1	12.3	8.5	6.6	(13-17 gm/dL)
MCV (fL)	83	82	84	85	85	85	84	87	88	76.6	74	71	(83-101 fL)
MCH (pg)	28	27	27	29	29	29	29	29	28	23	23.5	24	(27-32 pg)
Platelet (x10^3^/uL)	157	130	169	196	346	640	77	25	106	175	26	130	(150-410 x10^3^/uL)
Serum potassium K (mmol/L)	4.1	4.3	3.9	4.2	4.8	4.8	3.6	4	3.9	3	4.5	6.3	(3.5-5.3)
Serum sodium (mmol/L)	138	140	135	139	134	131	135	142	143	138	135	132	(133-146)
Serum calcium (mmol/L)	2.4	--	--	2.4	2.49	2.4	1.92	1.99	--	2.25	--	--	(2.2-2.6)
Serum magnesium (mmol/L)	0.36	0.8	0.84	--	--	--	0.7	--	--	0.38	0.89	1.19	(0.7-1)
Serum urea (mmol/L)	5.1	56	4.7	7.4	5.5	6.2	22	11.3	12.2	9.7	21.6	4.9	(2.5-7.8)
Serum creatinine (umol/L)	114	65	64	67	55	57	419	121	79	374	330	496	(62-106)
Serum albumin (gm/L)	27	22	26	23	20	22	27	22	--	28	27	25	(35-50)
Serum total protein (gm/L)	63	67	68	--	--	--	59	56	--	63	61	76	(60-80)
AST (IU/L)	16	26	22	--	--	--	263	207	--	74	285	114	(0-41)
ALT (IU/L)	31	33	54	--	--	--	86	72	--	45	153	85	(0-41)
Alkaline phosphatase (U/L)	67	310	150	--	--	--	74	178	--	147	179	227	(40–129)
Troponin T (ng/L)	2691	--	1064	--	--	--	--	--	--	297	4897	--	(3-15 ng/L)
Serum total bilirubin (mg/dl)	8	4	5	--	--	--	10	--	--	25	94	35	(0-21)
Serum chloride (mmol/L)	104	107	102	107	104	100	101	110	113	100	100	92	(95-108)
Serum bicarbonate (mmol/L)	17	26	25	25	22	22	17	27	25	13	23	18	(22-29)
CK (U/L)	--	--	--	--	--	--	10707	5587	--	--	3775	5905	(39-308 U/L)
Myoglobin (ng/mL)	--	33	--	--	--	--	7575	1019	--	--	9946	12469	(28-72 ng/mL)
CRP (mg/L)	274	141	69	336	277	259	334	433	225	133	427	196	(0-5 mg/L)
Procalcitonin (ng/mL)	>100	73			0.91		>100	95	33	>100	>100	>100	(<0.05 ng/mL)
PT (seconds)	22	11.7	14	15	--	--	18	11	12.3	19	13.4	14.2	(9.4-12.5 seconds)
INR	1.9	1	1.2	1.3	--	--	1.5	1.1	1.2	1.7	1.2	1.2	<1
D-Dimer (mg/L)	5.9	--	--	--	--	--	--	>35	--	--	19	5.7	(0-0.49 mg/L)
APTT (seconds)	37.9	73	37	33	--	--	46	39	27.9	50	37	34	(25.1- 36.5 seconds)
Lactic acid (mmol/L)	7	--	--	--	--	--	2.9	1.3	1.5	9.8	3	0.9	(0.5-2.2 mmol/L)

**Figure 5:**
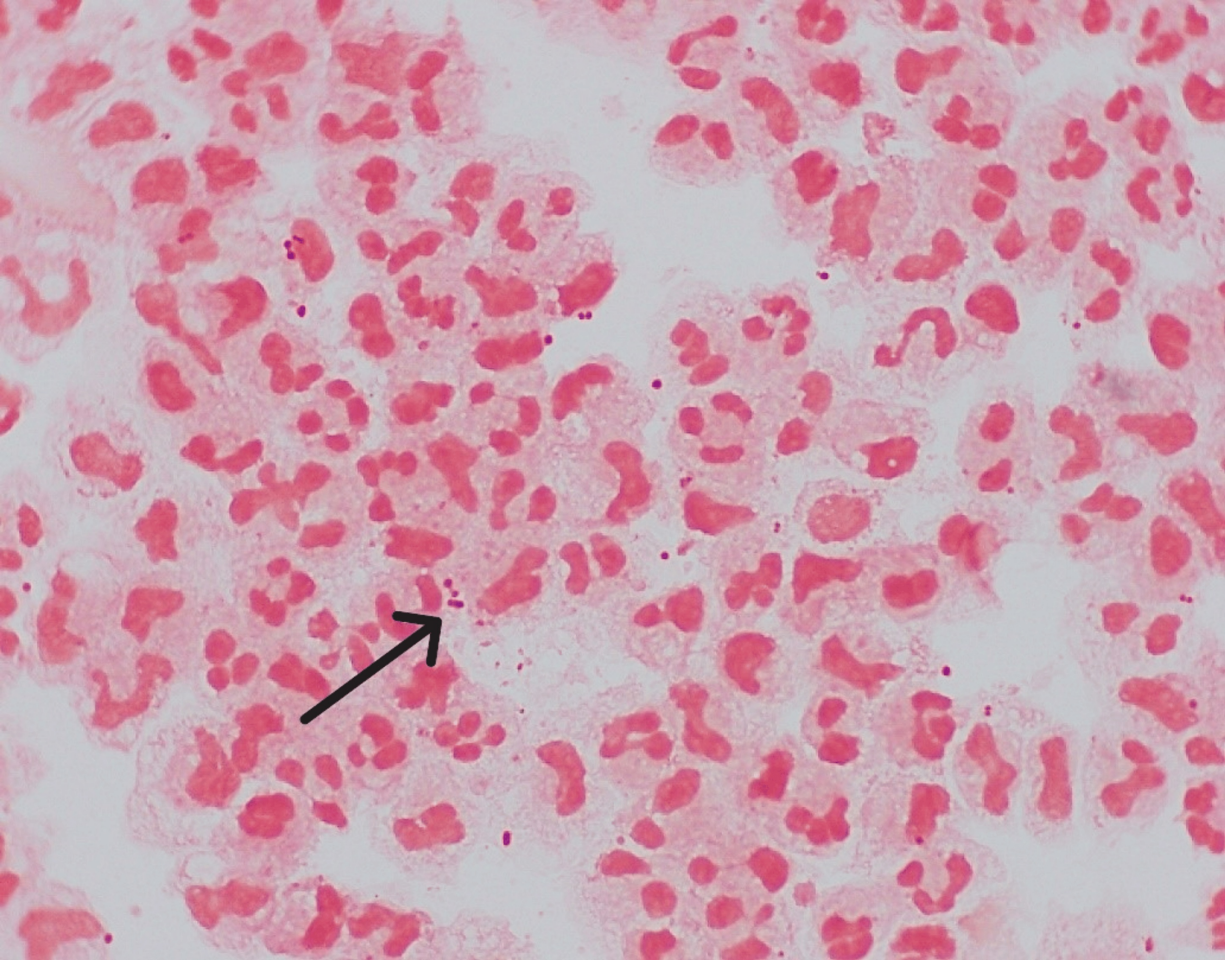
Direct gram stain on the CSF Gram negative diplococci in the cerebrospinal fluid (CSF)

A CT brain scan revealed no acute intracranial changes. A lumbar puncture was performed, and CSF analysis showed low glucose and high protein. CSF and blood cultures later confirmed *Neisseria meningitidis* serogroup W135 (Figure 5). The patient was diagnosed with severe meningococcal meningitis with meningococcemia, complicated by septic shock, acute kidney injury, rhabdomyolysis, and disseminated intravascular coagulation (DIC).

The patient was started on ceftriaxone (2 g twice daily) for meningococcal meningitis. Levetiracetam (Keppra) was continued with a loading dose of 4.5 g, followed by a maintenance dose of 1.5 g twice daily for seizure control. Aggressive fluid resuscitation was initiated for septic shock and acute kidney injury (AKI), and midazolam infusion was continued for sedation.

**Figure 6:**
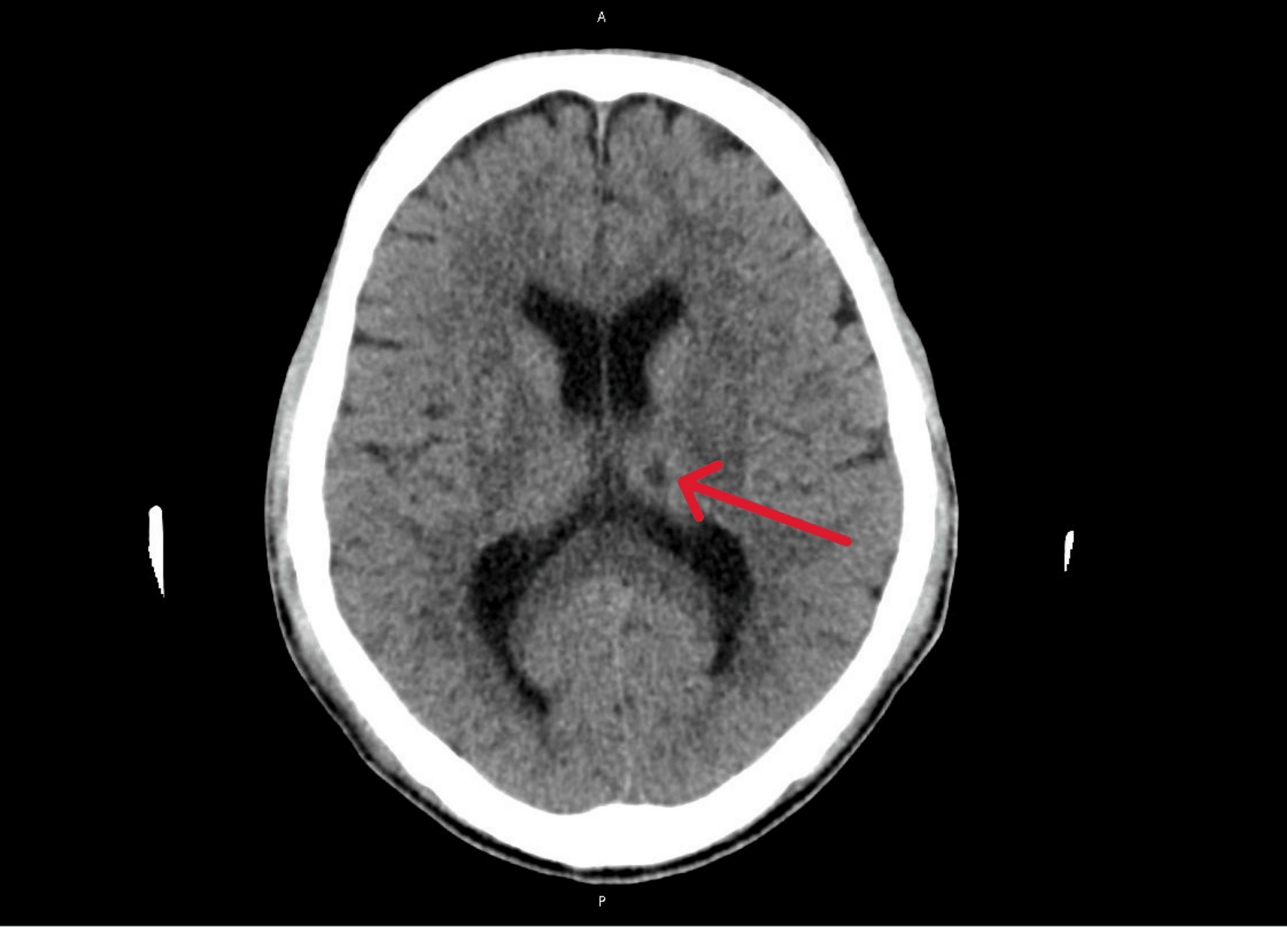
Focal hypodense area in the left thalamus

**Figure 7:**
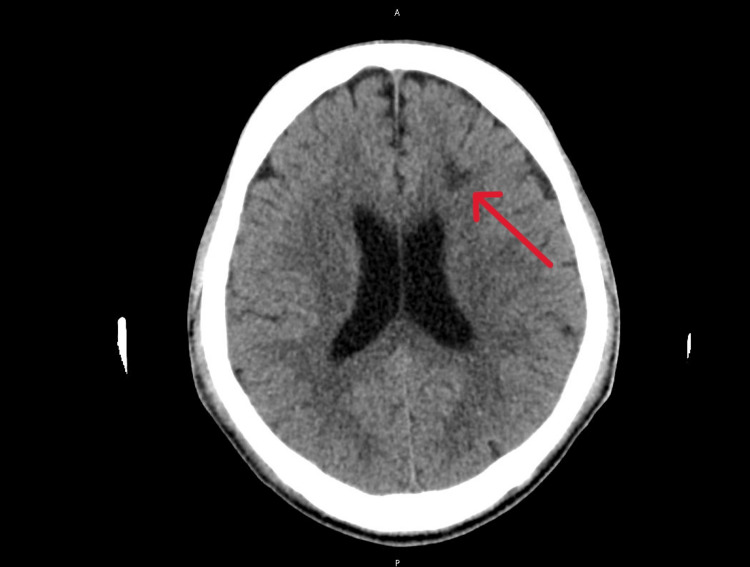
Focal hypodense area in the left frontal subcortical white matter

A repeated CT brain scan confirmed the presence of focal hypodense areas in the left thalamus (Figure 6) and the left frontal subcortical white matter (Figure 7). These likely represent recent ischemic lesions. He remains intubated and, therefore, cannot be assessed clinically.

The patient remained critically ill but showed gradual improvement with treatment. Renal parameters trended down, and septic shock was managed with vasopressors and fluid resuscitation. The patient was closely monitored in the ICU for complications, including DIC and rhabdomyolysis. He remains intubated but is hemodynamically stable, with vasopressors discontinued and inflammatory markers improving.

Case 3

A previously healthy 42-year-old male, who had not received the meningococcal vaccine, presented with acute-onset left shoulder pain and right-hand pain for two days, accompanied by fever. He denied any history of arthritis, skin rash, or trauma. The patient had previously visited the emergency room for cough and fever. On admission, he was afebrile but tachycardic (heart rate 124 bpm) and normotensive (blood pressure 120/75 mmHg). Physical examination revealed tenderness and mild swelling in the left shoulder, with limited active and passive range of motion. The right hand showed swelling and redness, consistent with cellulitis. A macular rash was noted on the dorsum of the right hand and the left upper arm. Neurological examination was normal, with no motor or sensory deficits. Cardiovascular, respiratory, and abdominal examinations were unremarkable.

**Figure 8:**
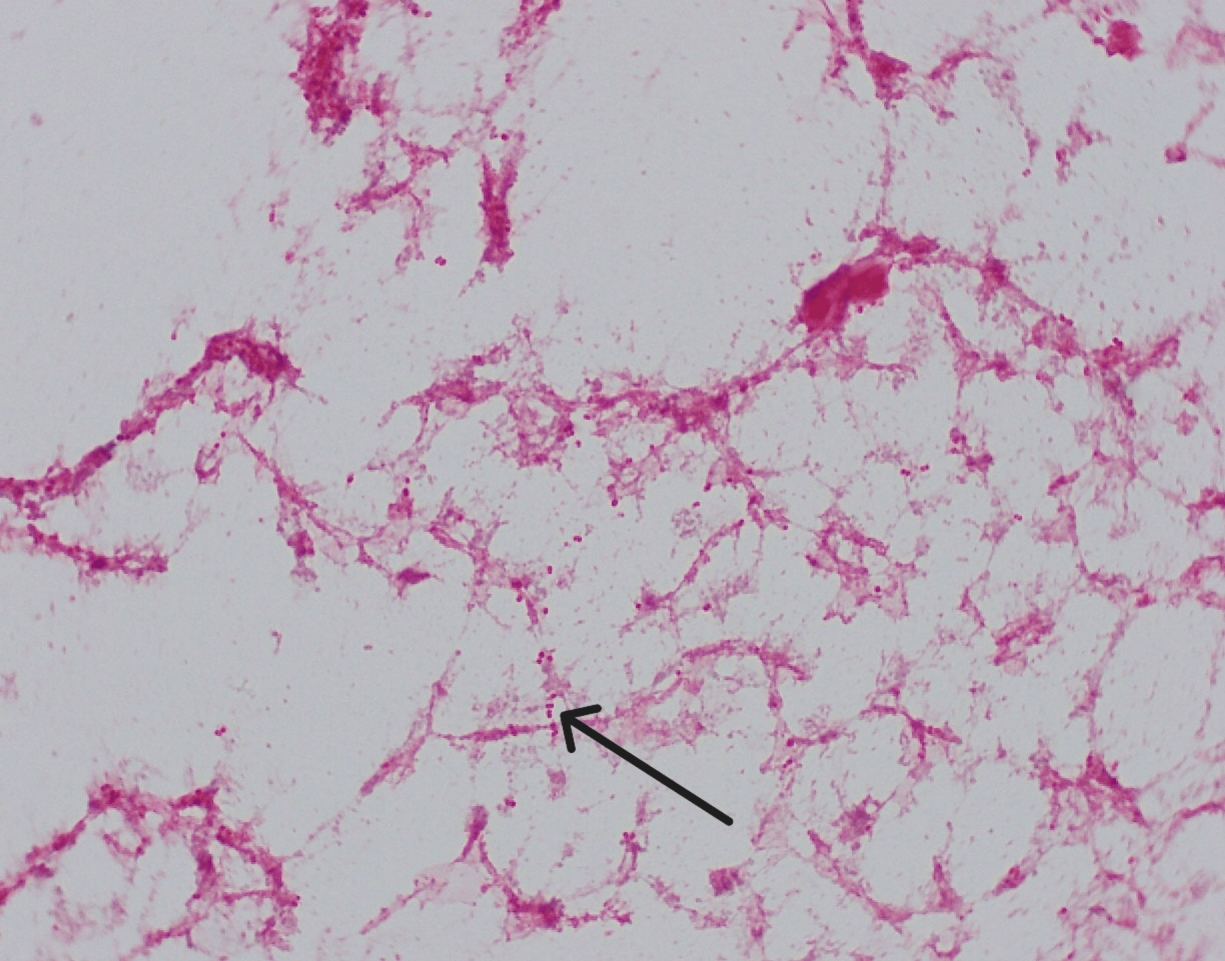
Gram negative diplococci from direct gram stain of the blood culture positive

Initial laboratory investigations revealed an increased WBC count, an increased CRP, and mildly deranged liver function tests. The patient was admitted as a case of cellulitis with polyarthritis and suspected meningitis. He was started on ceftriaxone (2 g every 12 hours). Blood cultures later confirmed *N. meningitidis* serogroup W135 (Figure 8), while cerebrospinal fluid (CSF) culture was negative. The patient was diagnosed with meningococcemia with secondary cellulitis and polyarthritis, with no evidence of meningitis. Polyarthritis and shoulder effusion were attributed to systemic infection rather than primary septic arthritis.

**Figure 9:**
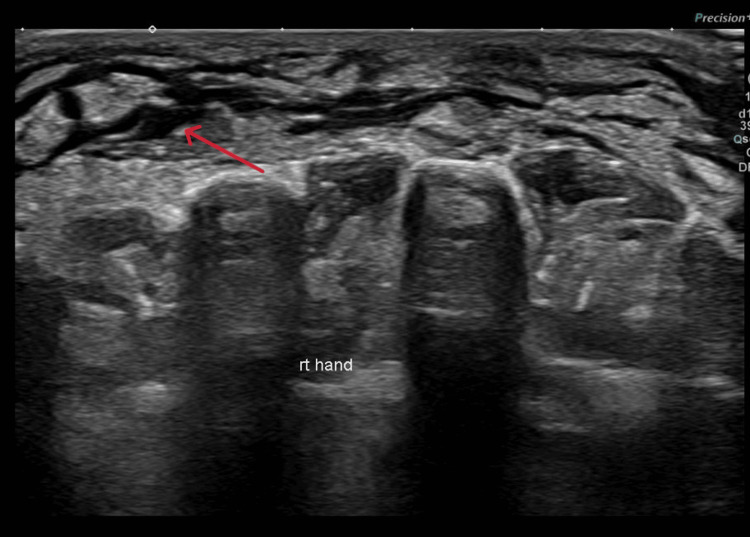
Edematous soft tissue swelling without collection

An ultrasound of the left shoulder revealed minimal fluid along the biceps tendon and posterior joint recess, suggesting minimal effusion or bursitis. Ultrasound of the soft tissue over the cellulitis area of the right hand revealed edematous soft tissue swelling on the dorsal aspect without collection (Figure 9). The orthopedic and rheumatology teams were consulted due to concerns about septic arthritis. However, imaging and clinical findings did not support a diagnosis of septic arthritis, and no surgical intervention was required. The patient’s condition was attributed to systemic meningococcemia with secondary cellulitis and polyarthritis.

Throughout his hospitalization, the patient showed gradual improvement with antibiotic therapy. By day four, he reported feeling much better, although mild pain, swelling, and redness persisted in the right hand and left shoulder. Range of motion in the left shoulder remained limited. Inflammatory markers trended downward, with CRP decreasing to 267 mg/L. The patient remained hemodynamically stable, with improving clinical symptoms. He remains on antibiotic therapy, with a planned 14-day treatment duration and a scheduled follow-up appointment.

Case 4

A previously healthy 23-year-old male, who had not received the meningococcal vaccine, presented with fever, epigastric pain, lower limb pain, knee pain, and burning micturition. His fever had started the night before admission. In the ED, he was found to be hypotensive, tachycardic, and tachypneic, with metabolic acidosis and elevated lactate. Fluid resuscitation was initiated with 2 liters of IV fluids. He initially responded but later became hypotensive again. Non-invasive ventilation (NIV) and noradrenaline were initiated; however, due to persistent hypotension, he was admitted to the ICU.

On arrival at the ICU, the patient was fully conscious but hypotensive and tachycardic, requiring noradrenaline at 0.5 mcg/kg/min. He was on NIV with 40% FiO_2_, maintaining adequate oxygen saturation. He was anuric and had severe knee pain, with patchy cyanosis of the skin on the lower limbs. A central line and arterial line were inserted, and he received additional fluid resuscitation with plasma protein fraction (PPF) and Ringer’s lactate. Vancomycin (1g) was administered empirically. The patient was deteriorated and intubated.

**Figure 10:**
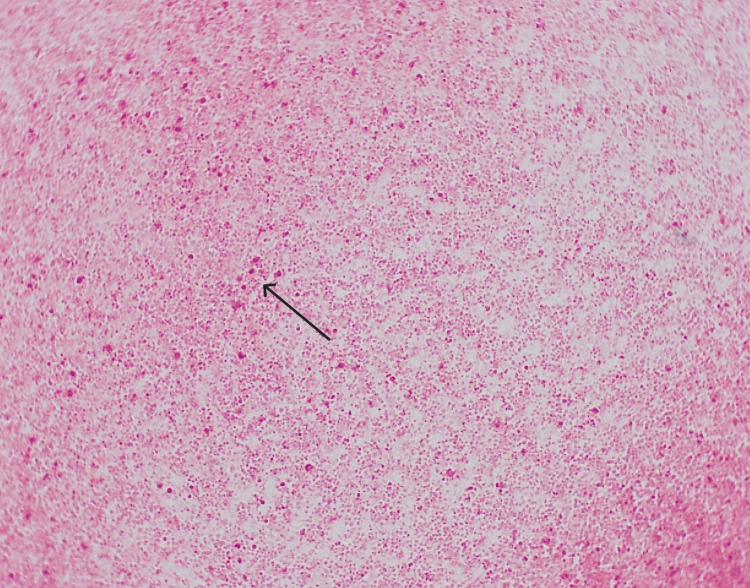
N. meningitidis colonies on blood agar are grayish, nonhemolytic, round, convex, smooth, moist, and glistening with a clearly defined edge

Initial laboratory investigations revealed increased lactate, CRP, and markedly increased procalcitonin. Acute kidney injury was evident with increased creatinine and urea levels. Liver enzymes were increased, and bilirubin was elevated. Markers of muscle injury were significantly increased, consistent with rhabdomyolysis. Hematology revealed leukocytosis, anemia, and thrombocytopenia (Table 1). Chest X-ray showed bilateral consolidations in the lower zones, consistent with pneumonia or acute respiratory distress syndrome (ARDS). Blood cultures grew Gram-negative diplococci, later called *Neisseria meningitidis* (Figure 10). Respiratory viral panel, influenza A/B, COVID-19, and legionella tests were negative. Hepatitis B/C and HIV screening were non-reactive.

The patient was diagnosed with meningococcemia complicated by septic shock, acute respiratory distress syndrome (ARDS), and multiorgan failure. He was started on meropenem (2g q8h) and linezolid 600mg q12h for broad-spectrum coverage. Despite aggressive management, his condition deteriorated, and he was intubated on the second day of admission due to worsening respiratory failure. He required prone ventilation and continuous renal replacement therapy (CRRT) for anuric AKI. Cardiology was consulted for ST-segment elevation on ECG and elevated troponin (40,134 ng/L). Initial echocardiography revealed an ejection fraction of 35% with diffuse hypokinesia, consistent with septic cardiomyopathy. A repeat echocardiogram showed improvement in EF to 50%, with no evidence of myocardial infarction. The cardiac team attributed the findings to septic shock and myocarditis.

Throughout his hospitalization, the patient remained critically ill but showed gradual improvement. By day five, he was hemodynamically stable and off vasopressors, with downward inflammatory markers. He continued on mechanical ventilation with permissive hypercapnia (target pH >7.2) and CRRT for AKI. Antibiotics were tapered to ceftriaxone (2 g twice daily) based on blood culture sensitivities. The diagnosis was confirmed as meningococcemia with secondary septic shock, ARDS, myocarditis, rhabdomyolysis, and multiorgan failure. He remains under close monitoring for further complications. He was extubated on day 19 after admission and stepped down to the medical ward. He remains in the hospital in the rehabilitation stage.

## Discussion

Meningococcal disease was first described in 1805 during a meningitis epidemic in Geneva. The causative pathogen was isolated in 1882, and its asymptomatic carriage in the nasopharynx was recognized in 1890. In 1909, distinct serotypes were identified, leading to the development of serum therapy by Flexner in 1913 [1]. *N. meningitidis* is a gram-negative, facultatively anaerobic diplococcus identified by its oxidase activity and ability to oxidize glucose and maltose, distinguishing it from gonococci [2]. Based on capsular polysaccharides, it is classified into eight major serogroups (A, B, C, X, Y, Z, W135, and L) [2]. Serogroup W135, particularly the ST-11 clonal complex, has been associated with hypervirulence and severe disease, including outbreaks among Hajj and Umrah pilgrims. Rapid diagnosis is critical, with coagglutination assays and PCR aiding early detection, especially in the CSF.

Between 2006 and 2015, meningococcal disease incidence in the U.S. dropped by 78%, with a shift in the median age of patients from 17 to 42 years [3]. The incidence of serogroups B, C, and Y declined, while serogroup W remained stable. However, cases of serogroup W ST-11 infections, particularly in adults, raised concerns [4]. The increasing prevalence of serogroup W135 in regions like Saudi Arabia and sub-Saharan Africa highlights the need for targeted vaccination strategies, particularly for travelers and high-risk populations. The highest fatality rates were linked to serogroups W and C. Infants and elderly patients had the highest incidence and mortality rates. Males and African Americans had slightly higher incidence rates. Meningococcal infections peaked in February and March, with serogroups B, C, and Y causing most cases, particularly among older children and adults [5].

Meningococcal epidemics caused by *N. meningitidis* serogroup A are frequent in regions like sub-Saharan Africa, Asia, and South America, occurring every 7 to 10 years [6], with case rates reaching 1 in 1,000 people and 1 in 100 for children under two years. A major outbreak of serogroup C occurred in sub-Saharan Africa in 2015, prompting WHO surveillance and vaccination strategies, which have proven effective in controlling epidemics [7]. The 2016-2017 serogroup C epidemic in Nigeria, with over 14,000 suspected cases, also led to successful vaccination efforts [8]. Serogroup W, though less common but more likely to cause meningococcemia than serogroup A, caused a significant outbreak among pilgrims returning from the Hajj in 2000-2001, and has been linked to endemic infections, particularly in South Africa, where its prevalence surged from 7% to 75% between 2000 and 2005 [9-10].

The exact cause of the epidemic spread of meningococcus remains unclear. However, it is considered a respiratory pathogen and is likely transmitted through aerosols. High attack rates in less developed countries are attributed to factors like poverty, crowding, poor sanitation, and malnutrition. Other contributing factors include herd immunity and specific virulence properties of epidemic strains [11]. Clonal analysis suggests that a strain from Central Asia spread through India, Pakistan, and Saudi Arabia to Africa via pilgrims from Mecca [12-13]. The nasopharyngeal carrier state is crucial in the transmission and development of disease. Epidemics in American military camps before vaccination support this idea, and the introduction of vaccination has prevented epidemics since 1972 [14].

*N. meningitidis* exclusively infects humans, with its pathogenesis linked to nasopharyngeal colonization and several virulence factors. Colonization of the nasopharynx, necessary for systemic infection, occurs through inhalation of aerosolized meningococci that attach to the epithelial surface via adhesion proteins, leading to invasion. Most carrier strains lack a capsule, which may result from downregulation or phase variation of capsule genes, enabling efficient adhesion and microcolony formation via adhesins like type IV pili [15]. While strains from infected patients belong to a few clonal types, carrier strains are more diverse, with only a small percentage being invasive clones [4]. Key virulence factors aiding adhesion, invasion, and immune evasion include pili, opacity proteins, lipooligosaccharides, capsular polysaccharides, and factor H-binding proteins [16].

Certain conditions increase susceptibility to meningococcal infection, such as complement deficiencies. Studies show that patients with terminal complement or properdin deficiencies are more likely to develop Neisserial disease, with a milder course and lower mortality compared to other populations [17-18]. The use of eculizumab, a monoclonal antibody that inhibits terminal complement, has been associated with a significantly increased incidence of meningococcal disease, sometimes leading to fatal infections [19]. HIV-infected individuals are also at a higher risk for invasive meningococcal disease, particularly in the antiretroviral therapy era, with one study showing a relative risk of 10 [20]. The risk is particularly elevated among those with a CD4^+^ count below 200 [20]. Additionally, men who have sex with men (MSM) have an increased incidence of meningococcal disease, with reports of serogroup C outbreaks among MSM in New York city, where the incidence rate was significantly higher than among non-MSM males [21-22].

All four cases we reported involved *N. meningitidis* serogroup W135, but the clinical presentations and complications varied significantly. Case 1, a 30-year-old male, presented with meningococcemia, septic shock, acute myocarditis, and purpura fulminans, requiring vasopressors and broad-spectrum antibiotics. He improved with meropenem and supportive care. Case 2, a 27-year-old male, had meningococcal meningitis with meningococcemia, complicated by seizures, septic shock, rhabdomyolysis, and DIC, requiring intubation and aggressive ICU management. Case 3, a 42-year-old male, presented with meningococcemia, secondary cellulitis, and polyarthritis, showing gradual improvement with ceftriaxone and no life-threatening complications. Case 4, a 23-year-old male, had meningococcemia complicated by septic shock, ARDS, rhabdomyolysis, myocarditis, and multiorgan failure, requiring mechanical ventilation. Despite critical illness, all cases showed gradual improvement with appropriate treatment, highlighting the importance of early recognition, aggressive antibiotic therapy, and supportive care in managing meningococcal infections.

The laboratory data revealed several key trends across the cases. Inflammatory markers were significantly elevated in all cases, with CRP levels exceeding 300 mg/L in Cases 2, 3, and 4, and procalcitonin levels >100 ng/mL in Cases 1, 3, and 4, indicating severe systemic inflammation. Leukocytosis was prominent in Cases 1, 3, and 4, with white blood cell (WBC) counts reaching 56 × 10³/µL in Case 4. Organ dysfunction was evident in Cases 3 and 4, with elevated creatinine and urea indicating acute kidney injury. Cases 2 and 4 also showed significant rhabdomyolysis, with creatine kinase (CK) levels exceeding 10707 U/L. Coagulopathy was observed in Cases 1, 3, and 4, with thrombocytopenia, elevated D-dimer, and prolonged prothrombin time (PT)/international normalized ratio (INR), consistent with DIC. Cardiac involvement was notable in Cases 1, 3, and 4, with Troponin T levels >2000 ng/L, indicating myocarditis or myocardial injury.

All patients received broad-spectrum antibiotics, with ceftriaxone and meropenem being the mainstays of treatment. Supportive care, including vasopressors, fluid resuscitation, and mechanical ventilation, was critical in severe cases. For Case 1, the development of atrial fibrillation in acute myocarditis was managed with aspirin, rivaroxaban, and bisoprolol, highlighting the importance of cardiac monitoring in meningococcal myocarditis. Case 1 showed significant improvement by day four, with discontinuation of vasopressors and resolution of atrial fibrillation. Case 2 remained intubated but hemodynamically stable, with improving inflammatory markers. The ischemic lesions observed on the CT brain in Case 2 underscore the potential for long-term neurological sequelae in severe meningococcal meningitis. Case 3 had a milder course, with gradual improvement on antibiotics, though mild pain and swelling persisted. The absence of septic arthritis in Case 3, despite polyarthritis, highlights the importance of distinguishing systemic inflammatory responses from primary joint infections. Case 4 remained critically ill but stabilized with mechanical ventilation, gradually improving by day five (Table 2).

**Table 2:** Summary and comparison of the four cases AKI: Acute Kidney Injury, ARDS: Acute Respiratory Distress Syndrome, CRP: C-reactive protein, CRRT: Continuous Renal Replacement Therapy, NIV: non-invasive ventilation

Case	Age/Sex	Key Symptoms	Complications	Diagnosis	Management	Outcome
1	30, Male	Fever, myalgia, sore throat, nausea, vomiting, diarrhea, fatigue	Septic shock, acute myocarditis, purpura fulminans, atrial fibrillation, peripheral ischemia	Meningococcemia with myocarditis and septic shock	IV fluids, vasopressors (dopamine, noradrenaline), meropenem, dexamethasone, aspirin, rivaroxaban, bisoprolol	Improved, de-escalated to ceftriaxone, vasopressors discontinued
2	27, Male	Fever, weakness, seizures	Meningitis, meningococcemia, septic shock, rhabdomyolysis, AKI, ischemic brain lesions	Meningococcal meningitis with meningococcemia	Ceftriaxone, levetiracetam, midazolam infusion, aggressive fluid resuscitation	Remains intubated, hemodynamically stable, vasopressors discontinued
3	42, Male	Fever, left shoulder pain, right-hand pain	Cellulitis, polyarthritis	Meningococcemia with cellulitis and polyarthritis	Ceftriaxone, orthopedic/rheumatology consultation	Improved, CRP decreasing, continuing antibiotic therapy
4	23, Male	Fever, epigastric pain, knee pain, burning micturition	Septic shock, rhabdomyolysis, ARDS, myocarditis, multiorgan failure	Meningococcemia with septic shock and multiorgan failure	IV fluids, vasopressors, NIV, meropenem, linezolid, prone ventilation, CRRT	Gradual improvement, hemodynamically stable off vasopressors, remains on mechanical ventilation and CRRT

Treating meningococcal sepsis is a complex, multidisciplinary effort requiring intensive care, infectious disease management, and coagulation support. In a medical emergency, early administration of appropriate antibiotics significantly improves outcomes, and appropriate parenteral therapy can clear the CSF of meningococci in less than six hours [23-24]. If feasible, blood cultures should be obtained before starting antibiotics, but treatment should not be delayed for a lumbar puncture. While early antibiotic use may reduce the likelihood of a positive CSF culture, diagnosis can still be confirmed through pretreatment blood cultures, PCR, or other molecular tools. PCR-based methods are particularly valuable in cases where blood cultures are negative due to prior antibiotic administration.

Acute systemic meningococcal disease typically presents as meningitis, meningitis with meningococcemia, or meningococcemia without clinical signs of meningitis [25]. Additional risk factors included autoimmune diseases, chronic respiratory infections, hemophilia, and low household income. Meningitis caused by *Neisseria meningitidis* typically presents with a sudden onset of fever, nausea, vomiting, headache, decreased concentration, and myalgias in an otherwise healthy patient [26]. A study found that the classic triad of fever, neck stiffness, and altered mental status was present in 27% of patients, and 89% showed at least two of these four signs when the rash was included [26]. The disease progresses rapidly, transitioning from health to severe illness in just hours. Key early signs of sepsis include leg pain, cold extremities, and abnormal skin color, and over 50% of patients present with a petechial rash, which can progress to larger purpuric lesions [25-27]. The shock state is frequently dominant in the manifestations of meningococcal meningitis and is characterized by poor responsiveness, cyanosis, and acidosis. Adrenal insufficiency from Waterhouse-Friderichsen syndrome and DIC can further contribute to hypotension and other complications [28].

The pathogenesis of DIC in meningococcal sepsis is linked to high levels of circulating microparticles that promote clot formation. Endotoxin-mediated activation of the coagulation cascade and tissue factor release from endothelial cells play a central role in DIC development. Clinical signs of DIC include subcutaneous hemorrhages, gastric or gingival bleeding, and oozing at venipuncture sites. Purpura fulminans, a severe complication seen in 15-25% of patients, involves acute hemorrhage and necrosis due to vascular thrombosis and DIC. It starts with pain, erythema, and petechiae, progressing to necrosis with bullae formation [29]. Myocardial failure and pulmonary edema are also common complications, with increased interleukin-6 levels contributing to myocardial depression [30].

All available meningococcal vaccines are inactivated and include pentavalent, quadrivalent, and monovalent formulations. The pentavalent vaccine (MenABCWY, Penbraya) targets serogroups A, C, W, Y, and B, incorporating a lyophilized MenACWY component conjugated to tetanus toxoid [30]. Quadrivalent vaccines cover serogroups A, C, W, and Y and include conjugate vaccines with a tetanus toxoid carrier and unconjugated polysaccharide vaccines, which are available in limited international markets for outbreak response and travelers [30]. Monovalent vaccines target individual serogroups, including a polysaccharide-tetanus toxoid conjugate vaccine for serogroup A, two vaccine formulations for serogroup B, and conjugate vaccine formulations for serogroup C [30]. 

## Conclusions

These cases highlight the diverse clinical spectrum of meningococcal infection, ranging from mild cellulitis and polyarthritis to life-threatening septic shock, multiorgan failure, and myocarditis. Early recognition of severe complications, such as septic shock, DIC, and myocarditis, is crucial for timely intervention. Laboratory markers, including CRP, procalcitonin, creatinine, CK, and troponin T, are valuable for assessing disease severity and guiding treatment. Aggressive antibiotic therapy and supportive care are essential in severe cases, particularly those involving septic shock and multiorgan failure. Prognostic indicators, such as persistent thrombocytopenia, elevated D-dimer, and high lactate levels, are associated with poor outcomes and should be closely monitored. These findings underscore the importance of vaccination strategies, particularly for high-risk populations, to reduce the burden of meningococcal disease. Continued vigilance, early intervention, and targeted prevention efforts are essential to improving outcomes in this potentially devastating condition. 
